# Acute mania and psychosis potentially triggered by St John's wort

**DOI:** 10.1002/pcn5.70292

**Published:** 2026-01-23

**Authors:** Daisuke Yoshioka, Takehiko Yamanashi, Masaaki Iwata

**Affiliations:** ^1^ Department of Psychiatry Yowa Hospital Yonago Japan; ^2^ Department of Neuropsychiatry, Faculty of Medicine Tottori University Tottori Japan

**Keywords:** herbal supplement, *Hypericum perforatum*, mania, St John's wort, substance‐induced psychosis

## Abstract

**Background:**

St John's wort (SJW) is widely used as an herbal supplement for depressive symptoms and is generally regarded as safe. However, although extremely rare, manic and psychotic reactions have been reported, typically in individuals taking high doses, using psychotropic agents, or having a psychiatric history.

**Case Presentation:**

We report a case of a Japanese male in his early twenties with only one prior psychiatric visit for interpersonal stress, who developed acute manic and psychotic symptoms while taking standard doses of SJW. Shortly after returning to a university training program following COVID‐19 infection, he developed emotional instability, prominent grandiose and persecutory delusions, reduced sleep, and dangerous behavior. Blood tests, cerebrospinal fluid analysis, brain imaging, and cerebrospinal fluid antibody testing were normal. Upon admission, we provisionally diagnosed him with schizoaffective disorder and initiated treatment with aripiprazole, which led to rapid improvement and near remission within 1 week. During pre‐discharge psychoeducation, he reported taking SJW for several months, suggesting a possible association between SJW use and symptom onset. SJW was discontinued, and aripiprazole long‐acting injectable treatment was initiated after discharge. After 2 months of sustained clinical stability, antipsychotic treatment was discontinued based on shared decision‐making with the patient. Outpatient follow‐up is ongoing to monitor for any recurrence of symptoms.

**Conclusion:**

This case demonstrates that acute manic and psychotic symptoms may occur even in young adults with no notable psychiatric history who take standard doses of SJW. Although SJW is regarded as safe, this case highlights that severe psychiatric reactions may still arise.

## BACKGROUND

St John's wort (SJW; *Hypericum perforatum*) has traditionally been used for a variety of neurological and psychiatric conditions. Its antidepressant efficacy for mild to moderate major depressive disorder has been supported by numerous randomized controlled trials and several meta‐analyses, demonstrating effectiveness comparable to standard antidepressants.^1^, ^2^ In addition to its favorable tolerability and over‐the‐counter availability, such scientific evidence has further contributed to its continued use. SJW is now one of the most widely used herbal supplements for depressive symptoms, particularly in European countries such as Germany, where it remains among the leading treatments for mild to moderate depression.^3^

The pharmacological actions of SJW are multifaceted, involving inhibition of serotonin, norepinephrine, and dopamine reuptake, as well as modulatory effects on GABAergic and glutamatergic neurotransmission.^4^, ^5^, ^6^ While these multimodal mechanisms are thought to underlie its antidepressant properties, they also raise concerns regarding potential psychiatric adverse reactions. Although large‐scale studies have generally reported a favorable safety profile,^7^, ^8^ case reports have described rare but clinically significant psychiatric effects such as mania, psychosis, and serotonin toxicity.^9^, ^10^ Moreover, because SJW is often self‐administered without medical supervision, such adverse reactions may be overlooked or misattributed.

Given these considerations, clinicians should be aware that SJW may precipitate mood elevation or psychotic symptoms. Here, we report a case of a young adult male who developed acute manic and psychotic symptoms in the context of SJW use.

## CASE PRESENTATION

The patient was a Japanese male in his early twenties. His family history was notable for a maternal great‐grandmother who had died by suicide, although further details were unavailable. During the spring of his university years, he had a single outpatient psychiatric visit for interpersonal stress‐related symptoms, without receiving a psychiatric diagnosis, psychotropic treatment, or subsequent follow‐up. In the autumn of the same year, he began a university training program. Shortly thereafter, he contracted COVID‐19 but recovered within several days and returned to the program 10 days after symptom onset. A few days after his return, he was reprimanded by an instructor, and that evening, his family found him crying at home. The following day, he became talkative and emotionally unstable, but his family initially chose to monitor him without intervention. Over the next several days, his behavior during the training became increasingly inappropriate, and staff instructed him to return home. However, despite being told to go home, he returned to the facility shortly thereafter, where he was approached by police officers after being seen smoking in front of the building.

During this period, he exhibited marked grandiosity. For instance, he asserted that “this training program is made possible by me” and that he wished to “speak directly” with a famous singer. He also displayed persecutory delusions, such as asking his family whether a television station was secretly filming him. His sleep was reduced to 2–3 h per night, and he engaged in dangerous behavior, including going alone to the seaside in the middle of the night. Because of his pressured speech, grandiose and persecutory delusions, and markedly decreased sleep, he was brought to the psychiatric department of a university hospital.

At the initial hospital, the subacute onset following COVID‐19 infection raised concern for encephalitis‐related psychiatric symptoms. He was referred to neurology, where blood tests, cerebrospinal fluid analysis, and brain magnetic resonance imaging were performed, all of which showed no abnormalities, and encephalitis was considered unlikely. Because the psychiatric ward was at full capacity, he was transferred to our hospital on the same day. Subsequently, the results of cerebrospinal fluid antibody testing, including anti–*N*‐methyl‐d‐aspartate receptor antibodies, returned negative, thereby ruling out autoimmune encephalitis.

At the time of admission, the patient spoke rapidly and in a demanding manner, making statements such as “Turn off the television; it makes me angry,” “There is a listening device, isn't there?” and “There are no cameras in this room, right?” He also acknowledged experiencing auditory hallucinations. Both the patient and his family denied any illicit drug use. Based on the concurrent presence of prominent manic symptoms and psychotic symptoms within the same episode, we provisionally diagnosed him with schizoaffective disorder, and treatment with aripiprazole 24 mg/day was initiated the following day. During the first few days of treatment, he continued to show elevated mood and grandiose behavior, speaking about his romantic experiences and addressing staff in an authoritative manner. However, his symptoms improved rapidly, and within 1 week, he had nearly achieved remission. After discussing treatment options with the patient, we planned to transition him to long‐acting injectable (LAI) aripiprazole in the outpatient setting, and he was discharged after 3 weeks of hospitalization.

At the time of psychoeducation regarding his diagnosis before discharge, the patient reported that several weeks before his initial outpatient psychiatric visit in the spring, he had begun taking SJW supplements once or twice per week before meeting others. After the university training program began, he started taking a standard dose of SJW every day to cope with increased stress, and continued this daily use until the day before admission. The product contained 650 mg of SJW extract per day, including 1.95 mg of hypericin and 19.5 mg of hyperforin. Because the temporal association suggested a possible SJW‐induced manic and psychotic episode, we instructed him to discontinue SJW permanently. At the same time, schizoaffective disorder could not be ruled out. Therefore, we decided to continue antipsychotic treatment for the time being and planned to consider a supervised discontinuation trial once his clinical stability had been maintained for an adequate duration. At his first outpatient visit, aripiprazole LAI 400 mg was initiated. After 2 months of sustained clinical stability, we discussed with the patient the possibility that his episode had been induced by SJW and that continued antipsychotic treatment might not be necessary in such a case. Based on shared decision‐making, antipsychotic treatment was discontinued. He has remained free of symptom recurrence, and outpatient follow‐up is ongoing to monitor for any relapse.

## DISCUSSION

This case involved a young adult male who developed acute manic and psychotic symptoms while taking standard over‐the‐counter doses of SJW. The onset of psychiatric symptoms showed a close temporal association with ongoing SJW use, and the symptoms improved rapidly with antipsychotic treatment, suggesting that the episode was likely a substance‐induced manic and psychotic reaction triggered by SJW.

SJW is generally considered to have a favorable safety profile, and this has been demonstrated in systematic reviews. A pooled analysis of 35,562 individuals reported that the incidence of adverse events ranged from 0% to 5.7%, comparable to placebo.^7^ Similarly, a review of 16 post‐marketing surveillance studies found that SJW was approximately 10 times safer than synthetic antidepressants, with adverse event rates ranging from 0.1% to 2.4%.^8^ While several case reports have described the emergence of manic or psychotic symptoms associated with SJW use, many of these cases involved factors such as a pre‐existing psychiatric disorder, high‐dose SJW intake, or concomitant use of psychotropic or recreational substances.^11^ Therefore, reports of psychiatric adverse effects occurring in individuals without a prior notable psychiatric history and taking standard doses of SJW, as in the present case, are exceedingly rare.

Evidence regarding the safety of SJW in younger populations remains sparse. Only a few small open‐label trials have examined SJW in adolescents with depression, reporting clinical improvement and generally mild adverse effects.^12^, ^13^ However, these studies lacked placebo control, included small samples, and had high dropout rates, making it impossible to evaluate the risk of infrequent but serious psychiatric reactions. Although the widespread use of SJW in Europe and the United States suggests that severe adverse events are likely uncommon, the absence of randomized controlled trials in adolescents and young adults means that the psychiatric safety of SJW in late adolescence and early adulthood—when primary mood and psychotic disorders often first emerge—remains insufficiently characterized. Overall, the current evidence is inadequate to draw firm conclusions about the risk of rare psychiatric adverse events in younger individuals.

Given the limited evidence regarding the safety of SJW in adolescents and young adults, caution is warranted when this product is used by individuals in these age groups. The contents and concentrations of active constituents such as hypericin and hyperforin vary substantially across commercial SJW products, making it difficult to accurately assess the true dosage or exposure in clinical practice. Furthermore, patients often perceive dietary supplements as inherently safe and may not volunteer information about their use unless specifically asked. This case underscores the importance of routinely inquiring about supplement use during psychiatric evaluations, particularly in cases of new‐onset mood or psychotic symptoms.

This report has notable limitations. The temporal association between changes in SJW use and symptom onset, the prompt improvement of psychiatric symptoms, the absence of symptom recurrence after discontinuation of antipsychotic medication, and the lack of a notable prior psychiatric or family history support the possibility of an SJW‐induced psychiatric episode; however, this interpretation remains uncertain. Alternatively, psychosocial stress related to the university training program and the recent course of COVID‐19 infection may have played a contributory role in the onset of psychiatric symptoms. Furthermore, it is possible that SJW did not act as a direct cause but rather served as a trigger for the first manifestation of an underlying mood or psychotic disorder. Long‐term follow‐up will therefore be essential to clarify the patient's diagnostic course. Another limitation of this report is that no objective substance screening, such as urine drug screening, was performed at either the referring hospital or our institution. Information regarding substance use was obtained solely through interviews with the patient and his family. Although substance use was considered unlikely based on these interviews, the absence of objective drug screening represents a limitation of this case.

## CONCLUSION

This case demonstrates that acute manic and psychotic symptoms can occur in a young adult with no notable psychiatric history who was taking only standard doses of SJW. Routine inquiry about supplement use is essential when assessing new‐onset psychiatric symptoms. Further evidence is needed to clarify the psychiatric risks of SJW in younger individuals.

## AUTHOR CONTRIBUTIONS

Daisuke Yoshioka treated the patient and drafted the manuscript. Takehiko Yamanashi and Masaaki Iwata critically reviewed the draft and revised it. All authors approved the final version of the manuscript.

## CONFLICT OF INTEREST STATEMENT

The authors declare no conflicts of interest.

## ETHICS APPROVAL STATEMENT

This study was conducted according to the principles of the Declaration of Helsinki.

## PATIENT CONSENT STATEMENT

Informed written consent and a signed release were obtained from the patient for the publication of this report.

## CLINICAL TRIAL REGISTRATION

Not applicable.

## Data Availability

Data sharing is not applicable to this article as no datasets were generated or analyzed during the current study.
